# Supplementary material for: Community’s misconception about COVID-19 and its associated factors in Satkhira, Bangladesh: A cross-sectional study
Source: PLoS One. 2021 Sep 10;16(9):e0257410. doi: 10.1371/journal.pone.0257410 (PMC8432838; doi:10.1371/journal.pone.0257410)
Supplement: Attachment — Submitted filename: PONE-D-21-10887_reviewer.pdf [file pone.0257410.s001.pdf]

PLOS ONE

Community's misconception about COVID-19 and its associated factors: Evidence from a cross-sectional study in Bangladesh

--Manuscript Draft--

**Manuscript Number:**	PONE-D-21-10887
**Article Type:**	Research Article
**Full Title:**	Community's misconception about COVID-19 and its associated factors: Evidence from a cross-sectional study in Bangladesh
**Short Title:**	COVID-19 related misconception in Bangladesh
**Corresponding Author:**	Md Nuruzzaman Khan<br>Jatiya Kabi Kazi Nazrul Islam University<br>Mymensingh, Bangladesh, BANGLADESH
**Keywords:**	Misconception; social and mass media; Rural area; COVID-19; Bangladesh
**Abstract:**	Introduction<br><br>Misconception about COVID-19 has been spread out broadly that the World Health Organization declared it as a major challenge in the fight against the disease. This study aimed to assess common misconceptions about COVID-19 among the rural people of Bangladesh and its associated socio-demographic and media related factors.<br><br>Methods<br><br>Data were collected from 210 respondents selected from three unions of Satkhira District, Bangladesh.The dependent variable was misconception about COVID-19 (Yes, No) which was generated based on the respondents' responses to six questions on common misconceptions of COVID-19. Explanatory variables were respondents' socio-demographic characteristics, mass media and social media use habits. Descriptive statistics were used to describe the characteristics of the respondents. Univariate and multivariate logistic regression models were used to determine the factors associated with misconception about COVID-19.<br><br>Results<br><br>Misconceptions about COVID-19 were found among more than half of the total respondents. More than 50% of the respondents reported they consider COVID-19 as a punishment from God. Besides, most of the respondents reported that they do not think COVID-19 is dangerous (59%), and COVID-19 is a disease (19%). Around 7% of the total respondents reported they consider this virus as a part of a virus war (7.2%). Bivariate analysis found that socio-demographic characteristics of the respondents and factors related to social and mass media were significantly associated with misconception. However, when all the factors included together in the multivariate model, the likelihood of misconception was lower among secondary (AOR, 0.33, 95% CI, 0.13-0.84) and tertiary (AOR, 0.29, 95% CI, 0.09-0.92) educated respondents compared to respondents with primary education.<br><br>Conclusion<br><br>This study obtained a very higher percentage of the rural people of Bangladesh having one or more misconceptions related to COVID-19. This could be a potential challenge in the fight against the pandemic. Effective use of mass and social media to communicate evidence based information on COVID-19 as well as to educate the public about COVID-19 is important.
**Order of Authors:**	Md. Bakebillah
	Md. Arif Billah
	Befikadu L. Wubishet

Powered by Editorial Manager® and ProduXion Manager® from Aries Systems Corporation

	Md Nuruzzaman Khan
**Additional Information:**	
**Question**	**Response**
**Financial Disclosure**<br><br>Enter a financial disclosure statement that describes the sources of funding for the work included in this submission. Review the submission guidelines for detailed requirements. View published research articles from *PLOS ONE* for specific examples.<br><br>This statement is required for submission and **will appear in the published article** if the submission is accepted. Please make sure it is accurate.<br><br>**Unfunded studies**<br>Enter: *The author(s) received no specific funding for this work.*<br><br>**Funded studies**<br>Enter a statement with the following details:<br>• Initials of the authors who received each award<br>• Grant numbers awarded to each author<br>• The full name of each funder<br>• URL of each funder website<br>• Did the sponsors or funders play any role in the study design, data collection and analysis, decision to publish, or preparation of the manuscript?<br>• **NO** - Include this sentence at the end of your statement: *The funders had no role in study design, data collection and analysis, decision to publish, or preparation of the manuscript.*<br>• **YES** - Specify the role(s) played.<br><br>* typeset	The author(s) received no specific funding for this work.
**Competing Interests**<br><br>Use the instructions below to enter a competing interest statement for this submission. On behalf of all authors, disclose any competing interests that could be perceived to bias this work—acknowledging all financial support and any other relevant financial or non-financial competing interests.	The authors have declared that no competing interests exist.

Powered by Editorial Manager® and ProduXion Manager® from Aries Systems Corporation

This statement is required for submission and **will appear in the published article** if the submission is accepted. Please make sure it is accurate and that any funding sources listed in your Funding Information later in the submission form are also declared in your Financial Disclosure statement.<br><br>View published research articles from *PLOS ONE* for specific examples.<br><br>**NO authors have competing interests**<br><br>Enter: *The authors have declared that no competing interests exist.*<br><br>**Authors with competing interests**<br><br>Enter competing interest details beginning with this statement:<br><br>*I have read the journal's policy and the authors of this manuscript have the following competing interests: [insert competing interests here]*<br><br>* typeset	
**Ethics Statement**<br><br>Enter an ethics statement for this submission. This statement is required if the study involved:<br><br>• Human participants<br>• Human specimens or tissue<br>• Vertebrate animals or cephalopods<br>• Vertebrate embryos or tissues<br>• Field research<br><br>Write "N/A" if the submission does not require an ethics statement.<br><br>General guidance is provided below. Consult the submission guidelines for detailed instructions. **Make sure that all**	Ethical approval was obtained from the Institute of Biological science of the University of Rajshahi.

information entered here is included in the Methods section of the manuscript.<br><br>**Format for specific study types**<br><br>**Human Subject Research (involving human participants and/or tissue)**<br>• Give the name of the institutional review board or ethics committee that approved the study<br>• Include the approval number and/or a statement indicating approval of this research<br>• Indicate the form of consent obtained (written/oral) or the reason that consent was not obtained (e.g. the data were analyzed anonymously)<br><br>**Animal Research (involving vertebrate animals, embryos or tissues)**<br>• Provide the name of the Institutional Animal Care and Use Committee (IACUC) or other relevant ethics board that reviewed the study protocol, and indicate whether they approved this research or granted a formal waiver of ethical approval<br>• Include an approval number if one was obtained<br>• If the study involved *non-human primates*, add *additional details* about animal welfare and steps taken to ameliorate suffering<br>• If anesthesia, euthanasia, or any kind of animal sacrifice is part of the study, include briefly which substances and/or methods were applied<br><br>**Field Research**<br><br>Include the following details if this study involves the collection of plant, animal, or other materials from a natural setting:<br>• Field permit number<br>• Name of the institution or relevant body that granted permission	
**Data Availability**<br><br>Authors are required to make all data underlying the findings described fully available, without restriction, and from the time of publication. PLOS allows rare exceptions to address legal and ethical	No - some restrictions will apply

concerns. See the PLOS Data Policy and FAQ for detailed information.<br><br>A Data Availability Statement describing where the data can be found is required at submission. Your answers to this question constitute the Data Availability Statement and **will be published in the article**, if accepted.<br><br>**Important:** Stating 'data available on request from the author' is not sufficient. If your data are only available upon request, select 'No' for the first question and explain your exceptional situation in the text box.<br><br>Do the authors confirm that all data underlying the findings described in their manuscript are fully available without restriction?	
**Describe where the data may be found in full sentences. If you are copying our sample text, replace any instances of XXX with the appropriate details.**<br><br>• If the data are **held or will be held in a public repository**, include URLs, accession numbers or DOIs. If this information will only be available after acceptance, indicate this by ticking the box below. For example: *All XXX files are available from the XXX database (accession number(s) XXX, XXX.*).<br>• If the data are all contained **within the manuscript and/or Supporting Information files**, enter the following: *All relevant data are within the manuscript and its Supporting Information files.*<br>• If neither of these applies but you are able to provide **details of access elsewhere**, with or without limitations, please do so. For example:<br><br>*Data cannot be shared publicly because of [XXX]. Data are available from the XXX Institutional Data Access / Ethics Committee (contact via XXX) for researchers who meet the criteria for access to confidential data.*	Data will be made available upon reasonable request to the corresponding author.

The data underlying the results presented in the study are available from (include the name of the third party and contact information or URL).<br>• This text is appropriate if the data are owned by a third party and authors do not have permission to share the data.<br><br>* typeset	
Additional data availability information:	

Main manuscript file Click here to access/download;Manuscript;Manuscript.docx

Title page

Community's misconception about COVID-19 and its associated factors: Evidence from a cross-sectional study in Bangladesh

Author(s): Md. Bakebillah[1#], Md. Arif Billah[2#], Befikadu L. Wubishet[3], Md. Nuruzzaman Khan[4*]

# Contributed equally to this manuscript

Affiliation(s)

[1]Department of Folklore,
Faculty of Social Science
Jatiya Kabi Kazi Nazrul Islam University
Trishal-2224, Mymensingh, Bangladesh

[2]Department of Psychology and Counselling
Faculty of Business, Economic and Social Development
Universiti Malaysia Terengganu
21030 Kuala Nerus, Terengganu, Malaysia

[3]Postdoctoral Research Fellow
Health Services Research Centre
Faculty of Medicine
The University of Queensland
Brisbane Qld 4072 Australia

[4]Department of Population Science
Faculty of Social Science
Jatiya Kabi Kazi Najrul Islam University
Trishal-2224, Mymensingh, Bangladesh

*Corresponding author

Short title: COVID-19 related misconception in Bangladesh

Abbreviations

COVID-19	Coronavirus Disease 2019
AOR	Adjusted Odds Ratio
OR	Odds ratio
SE	Standard Error
CI	Confidence Interval
HIV/AIDS	Human Immuno-deficiency Virus/ Acquired Immunodeficiency Syndrome
WHO	World Health Organization

Community's misconception about COVID-19 and its associated factors: Evidence from a cross-sectional study in Bangladesh

Abstract

Introduction: Misconception about COVID-19 has been spread out broadly that the World Health Organization declared it as a major challenge in the fight against the disease. This study aimed to assess common misconceptions about COVID-19 among the rural people of Bangladesh and its associated socio-demographic and media related factors.

Methods: Data were collected from 210 respondents selected from three unions of Satkhira District, Bangladesh.The dependent variable was misconception about COVID-19 (Yes, No) which was generated based on the respondents' responses to six questions on common misconceptions of COVID-19. Explanatory variables were respondents' socio-demographic characteristics, mass media and social media use habits. Descriptive statistics were used to describe the characteristics of the respondents. Univariate and multivariate logistic regression models were used to determine the factors associated with misconception about COVID-19.

Results: Misconceptions about COVID-19 were found among more than half of the total respondents. More than 50% of the respondents reported they consider COVID-19 as a punishment from God. Besides, most of the respondents reported that they do not think COVID-19 is dangerous (59%), and COVID-19 is a disease (19%). Around 7% of the total respondents reported they consider this virus as a part of a virus war (7.2%). Bivariate analysis found that socio-demographic characteristics of the respondents and factors related to social and mass media were significantly associated with misconception. However, when all the factors included together in the multivariate model, the likelihood of misconception was lower among secondary (AOR, 0.33, 95% CI, 0.13-0.84) and tertiary (AOR, 0.29, 95% CI, 0.09-0.92) educated respondents compared to respondents with primary education.

Conclusion: This study obtained a very higher percentage of the rural people of Bangladesh having one or more misconceptions related to COVID-19. This could be a potential challenge in the fight against the pandemic. Effective use of mass and social media to communicate evidence based information on COVID-19 as well as to educate the public about COVID-19 is important.

Keywords: Misconception, social and mass media, Rural area, COVID-19, Bangladesh

Background

Since December 2019, the world has been faced with the coronavirus outbreak which the World Health Organization later declared as a pandemic. All countries in the world are now affected by the pandemic and recorded high numbers of cases and deaths. Specially older people and individuals with pre-existing comorbidities such as diabetes and hypertension have been disproportionately affected by the pandemic. [1]. Similar to previous pandemics such as HIV/AIDS and Ebola, COVID-19 has been surrounded by a number of misconceptions from the very beginning [10,11] The existence of widely held misconceptions related to COVID-19 , particularly in the remote regions of developing countries including Bangladesh, has also been an important challenge compromising COVID-19 preventive efforts in communities [2,3]. For instance, many religious groups believe that this virus mainly affects non-religious communities or atheists while others believe educated and richer people are more prone to get the infection [3,4]. People in low-income countries have also been observed to believe that they have better immuity than people in developed countries which prevent them from getting COVID-19 [5,6]. There are other groups that believe coronavirus is a revenge of nature and punishment from God [7–9]. These misconceptions are dangerous because they deter disease preventive behaviours and also people with such misconceptions are less likely to report any cases and seek for health care [3,7,9]. The scarcity of evidence based information on both the virus and the disease as well as rapid emergence of these misinformation from numerous sources including social media are major reasons why the public dwelled in an "ocean" of misconceptions for so long [12]. Unfortunately misconeptions and misinformation are disseminated much quicker the correct information. The WHO defines this situation as '*infodemic'*, rapid and far-reaching spread of both accurate and

inaccurate information, which can potentially affect mental health and health related behavior of societies [12–14].

Bangladesh is one of the countries worst hit by the COVID-19 pandemic. More than a half million people has been infected among which around 9,000 people were died as per the official estimates of the World Health Organization for Bangladesh [15]. Given the low testing capacity of facilities and lack of accurate report of causes of deaths, these numbers could be reflecting underestimation of the real cases and deaths in the country, which is reflected in the reports of several private organizations and surveys [16,17]. Moreover, many Bangladeshi people have been observed to hold misconceptions about this virus, which makes them hide even if they have been infected or even death of a family member [16]. Another cause of possible underreporting of the true infection and death rates of this outbreak is social pressure. People mostly experience social isolation if they have been infected and therefore they prefer to hide their diagnosis. Importantly, these sorts of behaviors are common among people in rural and remote areas than their counterparts residing in urban areas [17–19].

There is a lack of study in Bangladesh on the issue of misconception about COVID-19. Only a few studies have been conducted but with several limitations including inappropriate list of misconceptions and inadequate sample [20,21]. Other focus areas of previous studies have been knowledge and awareness about COVID-19 among the general population [22], health professionals [23,24], parents [25] and students [26] and vulnerable and susceptible populations [27–29]. Moreover, very few studies have been conducted to explore misconceptions about COVID-19 that were disseminated through social and mass media [5,30,31]. Evidence from the

rural area is scarce till now. Therefore, this study aimed to explore the COVID-19 related misconception among the rural people of Bangladesh.

Methodology

Data source and sample

Primary data was collected during the period April 15 to May 09, 2020. Data was collected from three unions (smallest administrative classification of Bangladesh), Ratanpur, Kusulia and Moutala Union) of Satkhira district, Bangladesh. Multistage sampling was employed to select the unions and villages included in the study. At the first stage of the selection, Satkhira district was selected randomly from list of 64 districts in Bangladesh. These three unions have 68 villages [32]. We did a further random sampling to select 9 villages from these, three villages from each union. There were 113 households of the selected 9 villages of which 105 households were included in this survey. A fixed number of two members were included from each household with the inclusion criteria, (i) the youngest person aged more than 18 and (ii) the oldest person. The criteria were choosen to present family's most (over age 18) youngest and oldest person. This produced responses from 210 respondents which were our analysis sample for the study (Figure 1).

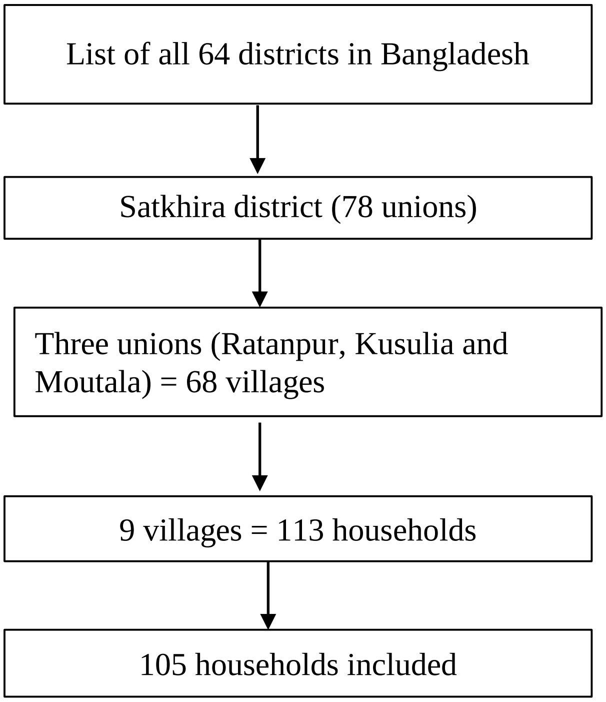

Figure 1. Sampling procedure for the study

Data collection

The questionnaire was first prepared in English and translated into Bengali, official language of Bangladesh. It was then back translated into English, to make sure the original meaning was retained. The questionnaire was developed based in the evidence of relevant research in Bangladesh and other developing countries [3,5,21,22,33,34]. Two rounds of pre-testing were conducted, and inaccuracies and misunderstood questions were revised before the final data collection. Verbal consent was taken from each respondent before collecting the data.

***Dependent* variable**

The dependent variable was misconception about COVID-19, that was created based on the respondent's response to six forms of misconception which were reported in Bangladesh at the time of the survey. These were COVID-19 is: (i) a punishment from god, (ii) revenge of nature, (iii) a virus war, (iv) not a dangerous virus, (v) not a disease and (vi) not a pandemic. Response to

each statement was reported dichotomously, “Yes” *or* “No”. We used these responses to generate a misconception variable with two categories, respondents had no misconception (0, if given correct response to all statements) and respondents had misconception (1, if given incorrect response to any of these statements).

Explanatory variables

The explanatory variables included were respondents’ socio-demographic characteristics and social and mass-media related practices. Socio-demographic characteristics included respondents’ gender, age, education and monthly income. Social and mass media related variables included respondents use of social media, daily frequency of social media use, reading newspapers, frequency of reading newspaper, watching television, frequency of watching television, listening to radio and frequency of listening to radio.

Analysis

Descriptive statistics were used to describe the characteristics of the respondents. Chi-square test was used to identify the variables significantly associated with misconception as well as their distribution. Bivariate and multivariate logistic regression models were used to identify the factors associated with misconception. The variables found significant in the chi-square analysis at 10% level of significance were included in the multivariate logistic regression model. Results were reported as Odd Ratio (OR) with 95% confidence interval (CI). The statistical software STATA version 14.3 was used for all statistical analyses.

Results

Characteristics of the study population

The characteristics of the respondents are presented in Table 1. Around two-thirds (66.19%) of the respondents were male. Most of the respondents (53.81%) were middle-aged (30-60 years) and

completed their secondary education (43.81%). The mean household income where the respondents resided in was 15,645 (SE, 758.123) Bangladeshi Taka (BDT). Most of the respondents lived in a small family (57.61%) and 67.14% had children in the family. About 87% of them reported use of mass media, of which 41% read newspapers, 82.4% watch television and 6.2% listen to the radio at least once in a week. Around 60% of the total respondents reported use of social media, more than a quarter of which using social media for more than two hours per day.

Table 1. Household characteristics of the study population

**Variables**	**Mean (SE)**	**Frequency**	**Percentage**
Gender			
Male		139	66.19
Female		71	33.81
Age group	***39.46 (1.115)***		
<30 years		67	31.90
30-59 years		113	53.81
60+ years		30	14.29
Education	***9.44 (0.290)***		
Primary		47	22.38
Secondary		92	43.81
Tertiary		71	33.81
Monthly family income	***15645.24 (758.123)***		
<=10000		102	48.57
>10000		108	51.43
Number of earning person in the family			
Only one		145	69.05
More than one		65	30.95
Family member	***4.55 (0.118)***		
Small family (<=4)		121	57.62
Extended family		89	42.38
Children in the family			
No		69	32.86
Yes		141	67.14
Older in the family			
No		135	64.29
Yes		75	35.71

Common misconception among the study population are presented in Table 2. More than half (52.86%) of the respondents reported one or more misconceptions. The most popular misconception in the study population was not considering the COVID-19 virus as dangerous (59%), followed by the belief that COVID-19 is a punishment from God (47%). There were also respondents who do not believe that the virus may cause a disease (19%) and some believe that COVID-19 is a virus war (7.6%).

Table 2: Common COVID-19 related misconceptions among rural people in Bangladesh, 2020

**Attributes**	**Frequency**	**Percentage**
COVID-19 is a punishment from God		
Have no misconception	111	52.9
Have misconception	99	47.1
COVID-19 is the revenge of nature		
Have no misconception	205	97.6
Have misconception	5	2.4
COVID-19 is a virus war		
Have no misconception	194	92.4
Have misconception	16	7.6
COVID-19 is a dangerous virus		
Have no misconception	87	41.1
Have misconception	123	58.6
COVID-19 is a disease		
Have no misconception	171	81.4
Have misconception	39	18.6
COVID-19 is a pandemic		
Have no misconception	209	99.5
Have misconception	1	0.5

The distribution of respondents with or without misconception across socio-demographic and media related factors is presented in Table 3. Misconception was reported higer among men (74.65%) than their women counterpart (25.25%). Lower level of misconception was found among the respondents who had secondary (47.47%) or tertiary (42.42%) level of education. About 70.27% respondents who did not read newspaper had high level of misconception whereas 87.88% respondents who watched television had low level of misconceptions about the COVID-19 disease.

Besides, those who use social media (72.73%) had a higher level of misconception regarding COVID-19 compared to the 52.25% misconnection among sample who reported they use social media. We found gender, monthly income, television use, treatment facilities, remedy measures were significantly associated with COVID-19 related misconception (p<0.05) in the bivariate analysis.

Table 3. Socio-demographic and media related variables among respondents with and without the COVID-19 related misconception

**Variable**	**COVID-19 related misconception**		**Chi-square/Fisher exact test (df), p-value**
	**No**	**Yes**	
Gender			***6.1286 (1), 0.013***
Male	74 (74.65)	65 (58.56)	
Female	25 (25.25)	46 (41.44)	
Education			***17.3052 (2), 0.000***
Primary	10 (1.11)	37 (33.33)	
Secondary	47 (47.47)	45 (40.54)	
Tertiary	42 (42.42)	29 (26.13)	
Monthly family income			***5.0016 (1), 0.025***
<=10000	40 (40.40)	62 (55.86)	
>10000	59 (59.60)	49 (44.14)	
Read Newspaper			***11.2862 (1), 0.001***
No	47 (47.47)	78 (70.27)	
Yes	52 (52.53)	33 (29.73)	
Watch Television			***3.9004 (1), 0.048***
No	12 (12.12)	25 (22.25)	
Yes	87 (87.88)	86 (77.48)	
Listen Radio			***2.7132 (1), 0.100***
No	90 (90.91)	107 (96.40)	
Yes	9 (9.09)	4 (3.60)	
Frequency of reading newspaper			***10.6437 (2), 0.005***
Not read	46 (46.46)	76 (68.47)	
Read but not daily	13 (13.13)	7 (6.31)	
Read daily	40 (40.40)	28 (25.23)	

Frequency of watching Television			***4.9216 (2), 0.085***
Not watch	12 (12.12)	26 (23.42)	
Watch but not daily	5 (5.05)	7 (6.31)	
Watch daily	82 (82.83)	78 (70.27)	
Frequency of listening radio			***3.0914 (2), 0.213***
Not listen	90 (90.91)	107 (96.40)	
Listen but not daily	5 (5.05)	3 (2.70)	
Listen daily	4 (4.04)	1 (0.90)	
Use social media			***13.5524 (1), 0.000***
No	27 (27.27)	58 (52.25)	
Yes	72 (72.73)	53 (47.75)	
Frequency of using social media/ day			***14.4197 (2), 0.001***
Don't use	27 (27.27)	58 (52.25)	
<=2 hrs	36 (36.36)	31 (27.93)	
>2 hrs	36 (36.36)	22 (19.82)	
Treatment facility			***7.5754 (2), 0.023***
No treatment	45 (45.45)	53 (47.75)	
Non-specialized treatment	16 (16.16)	32 (28.83)	
Specialized treatment	38 (38.38)	26 (23.42)	
Remedy			***6.8192 (2), 0.033***
Prayer	7 (7.07)	14 (12.61)	
Awareness	41 (41.41)	28 (25.23)	
Both	51 (51.52)	69 (62.16)	
Role of social media			***16.5407 (2), 0.000***
Negative	8 (8.08)	6 (5.41)	
Positive	78 (78.79)	63 (56.76)	
NA	13 (13.13)	42 (37.84)	

Results from the unadjusted and adjusted multivariate logistic regression analyses are presented in Table 4. Unadjusted logistic analysis found higher likelihood of misconception among women (OR, 2.1, 95% CI, 1.16 - 3.77) compared with men. Respondents who had reported their monthly income as more than 10000 BDT were 46% (OR, 0.54, 95% CI, 0.31 - 0.93) less likely to have misconception compared with those who reported their monthly income to be less than 10000 BDT (p<0.01). Compared with the primary educated respondents, those who attained secondary and tertiary level of education were 74% (05% CI, 0.12-0.58) and 81% (95%CI, 0.08-0.43) less likely to have misconception, respectively. Besides, the use of mass media (0.26, 0.10-67), and social

media ≤2 hrs per day (0.40, 0.21-0.78) and >2 hrs per day (0.28, 0.14-0.57) were found to be associated with 74%, and 60% and 72% lower level of misconception, respectively.

Adjusting the confounders, only education of the respondents had a significant association with misconception about COVID-19. Respondents having secondary and tertiary education were found 0.33 (95% CI, 0.13-84) and 0.29 (95% CI 0.09-0.92) less likely to report misconception about COVID-19 respectively than those having primary education. Importantly, the factors related to social and mass media were not as significant predictors of COVID-19 related misconceptions once respondent's characteristics were adjusted.

Table 4. Factors associated with the misconception about COVID-19 in Bangladesh

**Variables**	**Misconception of COVID-19**			
	**OR (SE)**	**95% CI**	**AOR (SE)**	**95% CI**
Gender				
Male	1		1	
Female	2.10 (0.631)**	1.1611-3.7791	1.64 (0.569)	0.8339-3.2376
Age group				
<30 years	1		1	
30-59 years	0.89 (0.274)	0.4829-1.6251	0.57 (0.216)	0.2738-1.2008
60+ years	0.81 (0.357)	0.3422-1.9211	0.56 (0.311)	0.1890-1.6643
Education				
Primary	1		1	
Secondary	0.26 (0.107)***	0.1152-0.5813	0.33 (0.157)*	0.1289-0.8398
Tertiary	0.19 (0.0803)***	0.0803-0.4339	0.29 (0.171)*	0.0924-0.9171
Monthly income				
<=10000	1		1	
>10000	0.54 (0.150)**	0.3094-0.9279	0.85 (0.288)	0.4347-1.6466
Use of Mass media				
No	1		1	
Yes	0.26 (0.126)**	0.1011-0.6737	0.77 (0.378)	0.2372-2.5120
Role of Mass media				
Negative	1		1	
Positive	0.89 (0.510)	0.2866-2.7393	0.61 (0.411)	0.1626-2.2849
NA/Others	2.57 (2.062)	0.5342-12.3781	0.75 (0.733)	0.1105-5.0945
Use of Social media				
No use	1		1	
<=2 hrs per day	0.40 (0.136)**	0.2066-0.7777	0.97 (0.453)	0.3893-2.4217
>2 hrs per day	0.28 (0.102)***	0.1413-0.5728	0.70 (0.378)	0.2495-2.0165
Role of social media				

Negative	1		1	
Positive	1.08 (0.610)	0.3551-3.2656	1.46 (0.961)	0.4000-5.3053
NA/others	4.31 (2.698)*	1.2619-14.7046	3.23 (2.438)	0.7372-14.1760
Treatment facility				
No treatment	1		1	
Non-specialized treatment	1.70 (0.624)	0.8268-3.4877	1.52 (0.642)	0.6608-3.4762
Specialized treatment	0.58 (0.189)*	0.3070-1.0992	0.94 (0.354)	0.4519-1.9691
Remedy				
Prayer	1		1	
Awareness	0.34 (0.179)**	0.1223-0.9533	0.55 (0.323)	0.1731-1.7394
Both	0.68 (0.337)	0.2547-1.7967	0.88 (0.489)	0.2957-2.6134
**Log likelihood= -124.85775, LR chi-square (17) = 40.72, p=0.0010 and Pseudo R-square= 0.1402**				

Note that- *p<0.05, **p<0.01, ***p<0.001. N=210

Discussion

The study identified common misconceptions related to COVID-19 in Bangladesh and their association with factors related to sociodemographic, mass and social media use of respondents. We found a high level of misconception about COVID-19 among our study sample which vary significantly across respondents' socio-demographic and media related factors. We also reported that the use of social and mass media and increase education were significantly related to the lower level of misconception about COVID-19 in rural Bangladesh. These findings are robust and could have an important policy implication to reduce the misconception over this pandemic in Bangladesh and other LMICs.

This study reported a very high level of misconception with around 52% of the total respondents. The commonest forms of misconception were this pandemic is a punishment of nature and the coronavirus (the virus that is known to cause COVID-19) is not a dangerous virus. These types of misconception were promoted by religious leaders and covered in several social and mass media [4,8]. Moreover, though it is established that COVID-19 is spill over infection and not a human made (laboratory generated) output [35–37], however, another common misconception is that this

virus is human created biological weapons for international politics [38]. Similar findings were reported across the world, including America [9,39], Saudi Arabia [31] Nigeria [33,40], Nepal [7], Ghana [41], Uganda [42] and Pakistan [43].

Socio-demographic characteristics of the respondents were associated with COVID-19 related misconceptions. For instance, COVID-19 related misconceptions are more common among people living in rural areas as reported in a previous study in Bangladesh [22]. People living in rural areas are usually have lower education and have limited access to the healthcare facilities and mass medias that could contribute to more widespread presence of such misconceptions. This is particularly true for the rural women of Bangladesh, as they are even more family-centric and most have lower education which is usually associated with higher infectious disease burden and related misconceptions. For instance, a significant proportion of the rural women believe children's respiratory disease (Acute respiratory diseases) was the influence of evils or supernatural beings, and therefore can only be treated through the spiritual healers [44,45]. Though these sorts of misconception are different from the misconceptions related to the COVID-19, however, their existence work as an easy root to spread out the COVID-19 related misconception.

Education plays a significant role to prevent infectious disease, like COVID-19, through better aware people creation about disease, education on causes of infection and ways of prevention. Educated people are usually have better access to the mass and social media which are the comments roots of spreading COVID-19 related misconceptions. However, educated people have the capacity to differentiate between the right and wrong. This enables them to recived the correct information from the mass and social media and avoid incorrect information which contribute to

the lowering misconceptions [43,46]. Another root of lower misconception about the COVID-19 among the educated people is access to the advanced medical treatment facilities (43).

The Government of Bangladesh is now using mass media to reduce misconceptions about the ongoing COVID-19 pandemic and disseminate correct information. The WHO recommends the same strategy to fight against this pandemic. For this, the newspapers, television channels have been broadcasting the regular updates about the spread of the disease, new findings, national and international strategies. However, access to mass media, except the radio, is significantly lower in rural area of Bangladesh compared to the urban area. The effectiveness of radio to disseminate such information has been questioned for a variety of reasons. For instance, radio often disseminate the one way of information, which is mostly difficult to understand for the rural and uneducated people. Moreover, such one way of information dissemination can have the little influence to justify and reduce misconception that the rural people get from other sources including social media where unauthentic information spread out rapidly. Additionally, it mostly fails to answer the questions that people have had regarding this disease, thorough this is an important way out to reduce misconception. Ensuring proper access to the mass media and dissemination of correct knowledge could therefore have a significant role to reduce misconception about COVID-19 pandemic.

This study has number of strength and limiatations. As far we know, this is the first study of its kind in Bangladesh that assess the misconception regarding the COVID-19 pandemic and associated factors. Moreover, this study consider a wide range of factors and identify their association with the COVID-19 related misconception. This makes this study findings is more

reliable. However, data collected was cross-sectional in nature, thereofore this study findings is correlational only, not causal. Moreover, this study data was collected from only one district and three unions, therefore they findings may not be geralizable for the whole population of Bangladesh though the district and unions included were selected randomly.

Conclusion

We found that many people living in rural parts of Bangladesh have one or more misconceptions about COVID-19. Respondents' socio-demographic characteristics and status of using mass and social media were identified as significant predictors of misconception related to COVID-19. Proper education and dissemination of correct information in mass and social media are important to reduce the misconceptions and succeed in the fight against this pandemic.

Conflict of interest: The authors declare that there is no conflict of interest.

Funding: There is no fund for this study.

Acknowledgement: We acknowledged to the respondents who had participated in this study voluntarily. We also give thanks to Mst. Sumaiya Sultana (Post Graduate student), Sk Mustafizur Rahman (physiotherapist), Prokash Patra (Science teacher at a high school), Ajgor Ali (Post graduate student), and Jayanta Kumar Ghosh (Village doctor) for being associated during the data collection at the field level. We also acknowledged the Department of Folklore, Faculty of Social Science, Jatiya Kabi Nazrul Islam University, Trishal-2224, Mymensingh, Bangladesh where this study is conducted.

References

1. Muniyappa R, Gubbi S. COVID-19 pandemic, coronaviruses, and diabetes mellitus. Am J Physiol-Endocrinol Metab. 2020;318: E736–E741. doi:10.1152/ajpendo.00124.2020

2. Brittany Linkous. Misconceptions and the Spread of Infectious Disease. In: Federation Of American Scientists [Internet]. 5 Jun 2014 [cited 6 Feb 2021]. Available: https://fas.org/pir-pubs/misconceptions-spread-infectious-disease/

3. Roy S. Low-income countries are more immune to COVID-19: A misconception. Indian J Med Sci. 2020;72: 5–7. doi:10.25259/IJMS_26_2020

4. Reuters. Islamic State calls COVID-19 God's punishment for foes: tape. Reuters. 28 May 2020. Available: https://www.reuters.com/article/us-health-coronavirus-islamicstate-idUSKBN2343B9. Accessed 6 Feb 2021.

5. Mekonnen HS, Azagew AW, Wubneh CA, Belay GM, Assimamaw NT, Agegnehu CD, et al. Community's misconception about COVID-19 and its associated factors among Gondar town residents, Northwest Ethiopia. Trop Med Health. 2020;48: 99. doi:10.1186/s41182-020-00279-8

6. Bates BR, Moncayo AL, Costales JA, Herrera-Cespedes CA, Grijalva MJ. Knowledge, Attitudes, and Practices Towards COVID-19 Among Ecuadorians During the Outbreak: An Online Cross-Sectional Survey. J Community Health. 2020;45: 1158–1167. doi:10.1007/s10900-020-00916-7

7. Dahal P. Covid: God may punish Nepal for cancelling rites, religious leaders warn. BBC News. 2 Oct 2020. Available: https://www.bbc.com/news/world-asia-54375436. Accessed 6 Feb 2021.

8. Kaur H. Ukrainian church leader who called Covid-19 "God's punishment" for same-sex marriage tests positive for virus. CNN. 9 Sep 2020. Available: https://www.cnn.com/2020/09/09/europe/ukrainian-leader-covid-same-sex-marriage-trnd/index.html. Accessed 6 Feb 2021.

9. Lofton K, Geneviève Z. If COVID-19 is a message from God, what's the message? In: courant.com [Internet]. 2020 [cited 6 Feb 2021]. Available: https://www.courant.com/opinion/op-ed/hc-op-lofton-beyerlein-nirenberg-zubrzycki-god-covid-0605-20200605-fijukv2szrezxixdol4b2ouw6e-story.html

10. Adongo PB, Tabong PT-N, Asampong E, Ansong J, Robalo M, Adanu RM. Beyond Knowledge and Awareness: Addressing Misconceptions in Ghana's Preparation towards an Outbreak of Ebola Virus Disease. Boni MF, editor. PLOS ONE. 2016;11: e0149627. doi:10.1371/journal.pone.0149627

11. Brooks RA, Allen VC, Regan R, Mutchler MG, Cervantes-Tadeo R, Lee S-J. HIV/AIDS conspiracy beliefs and intention to adopt preexposure prophylaxis among black men who have sex with men in Los Angeles. Int J STD AIDS. 2018;29: 375–381. doi:10.1177/0956462417727691

12. World Health Organization. Immunizing the public against misinformation. In: World Health Organization [Internet]. 10 Aug 2020 [cited 6 Feb 2021]. Available: https://www.who.int/news-room/feature-stories/detail/immunizing-the-public-against-misinformation

13. Gao J, Zheng P, Jia Y, Chen H, Mao Y, Chen S, et al. Mental health problems and social media exposure during COVID-19 outbreak. PLOS ONE. 2020;15: e0231924. doi:10.1371/journal.pone.0231924

14. Xiong J, Lipsitz O, Nasri F, Lui LMW, Gill H, Phan L, et al. Impact of COVID-19 pandemic on mental health in the general population: A systematic review. J Affect Disord. 2020;277: 55–64. doi:10.1016/j.jad.2020.08.001

15. Bangladesh: WHO Coronavirus Disease (COVID-19) Dashboard. [cited 30 Mar 2021]. Available: https://covid19.who.int

16. Anwar S, Nasrullah M, Hosen MJ. COVID-19 and Bangladesh: Challenges and How to Address Them. Front Public Health. 2020;8. doi:10.3389/fpubh.2020.00154

17. Bodrud-Doza M, Shammi M, Bahlman L, Islam ARMT, Rahman MM. Psychosocial and Socio-Economic Crisis in Bangladesh Due to COVID-19 Pandemic: A Perception-Based Assessment. Front Public Health. 2020;8. doi:10.3389/fpubh.2020.00341

18. Shammi M, Bodrud-Doza Md, Towfiqul Islam ARMd, Rahman MdM. COVID-19 pandemic, socioeconomic crisis and human stress in resource-limited settings: A case from Bangladesh. Heliyon. 2020;6: e04063. doi:10.1016/j.heliyon.2020.e04063

19. Ranscombe P. Rural areas at risk during COVID-19 pandemic. Lancet Infect Dis. 2020;20: 545. doi:10.1016/S1473-3099(20)30301-7

20. Islam AKMN, Laato S, Talukder S, Sutinen E. Misinformation sharing and social media fatigue during COVID-19: An affordance and cognitive load perspective. Technol Forecast Soc Change. 2020;159: 120201. doi:10.1016/j.techfore.2020.120201

21. Tasdik Hasan M, Hossain S, Saran TR, Ahmed HU. Addressing the COVID-19 related stigma and discrimination: A fight against "infodemic" in Bangladesh. Minerva Psichiatr. 2020;61: 184–187. doi:10.23736/S0391-1772.20.02088-9

22. Ferdous MZ, Islam MS, Sikder MT, Mosaddek ASM, Zegarra-Valdivia JA, Gozal D. Knowledge, attitude, and practice regarding COVID-19 outbreak in Bangladesh: An online-based cross-sectional study. PloS One. 2020;15: e0239254. doi:10.1371/journal.pone.0239254

23. Salman M, Mustafa Z, Asif N, Zaidi HA, Shehzadi N, Khan TM, et al. Knowledge, attitude and preventive practices related to COVID-19 among health professionals of Punjab province of Pakistan. J Infect Dev Ctries. 2020;14: 707–712. doi:10.3855/jidc.12878

24. Tamang N, Rai P, Dhungana S, Sherchan B, Shah B, Pyakurel P, et al. COVID-19: a National Survey on perceived level of knowledge, attitude and practice among frontline healthcare Workers in Nepal. BMC Public Health. 2020;20: 1905. doi:10.1186/s12889-020-10025-8

25. Abuhammad S. Parents' knowledge and attitude towards COVID- 19 in children: A Jordanian Study. Int J Clin Pract. 2020;75. doi:10.1111/ijcp.13671

26. Honarvar B, Lankarani KB, Kharmandar A, Shaygani F, Zahedroozgar M, Rahmanian Haghighi MR, et al. Knowledge, attitudes, risk perceptions, and practices of adults toward COVID-19: a population and field-based study from Iran. Int J Public Health. 2020; 1–9. doi:10.1007/s00038-020-01406-2

27. Iradukunda PG, Pierre G, Muhozi V, Denhere K, Dzinamarira T. Knowledge, Attitude, and Practice Towards COVID-19 Among People Living with HIV/AIDS in Kigali, Rwanda. J Community Health. 2020; 1–6. doi:10.1007/s10900-020-00938-1

28. Anikwe CC, Ogah CO, Anikwe IH, Okorochukwu BC, Ikeoha CC. Coronavirus disease 2019: Knowledge, attitude, and practice of pregnant women in a tertiary hospital in Abakaliki, southeast Nigeria. Int J Gynecol Obstet. 2020;151: 197–202. doi:10.1002/ijgo.13293

29. Akalu Y, Ayelign B, Molla MD. Knowledge, Attitude and Practice Towards COVID-19 Among Chronic Disease Patients at Addis Zemen Hospital, Northwest Ethiopia. Infect Drug Resist. 2020;Volume 13: 1949–1960. doi:10.2147/IDR.S258736

30. Early Herd Immunity against COVID-19: A Dangerous Misconception. In: Johns Hopkins Coronavirus Resource Center [Internet]. [cited 6 Feb 2021]. Available: https://coronavirus.jhu.edu/from-our-experts/early-herd-immunity-against-covid-19-a-dangerous-misconception

31. Baig M, Jameel T, Alzahrani SH, Mirza AA, Gazzaz ZJ, Ahmad T, et al. Predictors of misconceptions, knowledge, attitudes, and practices of COVID-19 pandemic among a sample of Saudi population. Menezes RG, editor. PLOS ONE. 2020;15: e0243526. doi:10.1371/journal.pone.0243526

32. সাতক্ষীরা জেলা. [cited 20 Mar 2021]. Available: http%3A%2F%2Fwww.satkhira.gov.bd%2Fsite%2Fpage%2F23cf9586-1c4b-11e7-8f57-286ed488c766%2F%25E0%25A6%258F%25E0%25A6%2595%2520%25E0%25A6%25A8%25E0%25A6%259C%25E0%25A6%25B0%25E0%25A7%2587%2520%25E0%25A6%25B8%25E0%25A6%25BE%25E0%25A6%25A4%25E0%25A6%2595%25E0%25A7%

258D%25E0%25A6%25B7%25E0%25A7%2580%25E0%25A6%25B0%25E0%25A6%25BE%2520%25E0%25A6%259C%25E0%25A7%2587%25E0%25A6%25B2%25E0%25A6%25BE

33. Aminu Julius A. The Implications of Misconceptions about Coronavirus Disease (COVID-19) Pandemic in Relation to its Daily Increases from Nigerian Perspective. J Infect Dis Epidemiol. 2020;6. doi:10.23937/2474-3658/1510156

34. Chepkwony AK, Etemesi IN. COVID-19: Re-Imagining of Beliefs and Religious Misinformation. Voices Pandemic Polycrisis Hopes. 2020;XLII: 27–34.

35. Nie J-B. In the Shadow of Biological Warfare: Conspiracy Theories on the Origins of COVID-19 and Enhancing Global Governance of Biosafety as a Matter of Urgency. J Bioethical Inq. 2020; 1–8. doi:10.1007/s11673-020-10025-8

36. Leite Júnior DP, Araújo Pires RA, Dantas ES de O, Pereira RS, Bonci MM, Ramos RTB, et al. Spillover: the role of bats and relationships as reservoirs of zoonotic viruses and the origin of new coronaviruses. Forensic Res Criminol Int J. 2020;8: 205–214. doi:10.15406/frcij.2020.08.00329

37. Kaszeta D. Perspective | No, the coronavirus is not a biological weapon. Washington Post. 27 Apr 2020. Available: https://www.washingtonpost.com/outlook/2020/04/26/no-coronavirus-is-not-biological-weapon/. Accessed 6 Feb 2021.

38. Imhoff R, Lamberty P. A Bioweapon or a Hoax? The Link Between Distinct Conspiracy Beliefs About the Coronavirus Disease (COVID-19) Outbreak and Pandemic Behavior. Soc Psychol Personal Sci. 2020;11: 1110–1118. doi:10.1177/1948550620934692

39. Malato D, Hernandez MM, Benz J, Bonnell W, Tompson T, Nirenberg D, et al. How Faith Shapes Feelings About the Coronavirus Outbreak. In: AP-NORC [Internet]. 2020 [cited 6 Feb 2021]. Available: https://apnorc.org/projects/how-faith-shapes-feelings-about-the-coronavirus-outbreak/

40. Isah MB, Abdulsalam M, Bello A, Ibrahim MI, Usman A, Nasir A, et al. Coronavirus Disease 2019 (COVID-19): Knowledge, attitudes, practices (KAP) and misconceptions in the general

population of Katsina State, Nigeria. medRxiv. 2020; 2020.06.11.20127936. doi:10.1101/2020.06.11.20127936

41. Saba CKS, Nzeh J, Addy F, Karikari AB. COVID-19: Knowledge, Perceptions and Attitudes of Residents in the Northern Region of Ghana, West Africa. 2020 [cited 6 Feb 2021]. doi:10.20944/preprints202008.0060.v1

42. Kasozi KI, MacLeod E, Ssempijja F, Mahero MW, Matama K, Musoke GH, et al. Misconceptions on COVID-19 Risk Among Ugandan Men: Results From a Rapid Exploratory Survey, April 2020. Front Public Health. 2020;8. doi:10.3389/fpubh.2020.00416

43. Khalid Z, Yousaf MA, Khan null AT-A, Shakoori FR, Munir M, Shakoori AR. Debunking myths about COVID-19, paranoiac misconceptions, recent developments and its current stance. Pak J Zool. 2020;52. doi:10.17582/JOURNAL.PJZ/20200608144416

44. Stewart MK, Parker B, Chakraborty J, Begum H. Acute respiratory infections (ARI) in rural Bangladesh: Perceptions and practices. Med Anthropol. 1993;15: 377–394. doi:10.1080/01459740.1994.9966100

45. Rashid SF, Hadi A, Afsana K, Begum SA. Acute respiratory infections in rural Bangladesh: cultural understandings, practices and the role of mothers and community health volunteers. Trop Med Int Health. 2001;6: 249–255. doi:https://doi.org/10.1046/j.1365-3156.2001.00702.x

46. Mondal MNI, Hoque N, Chowdhury MRK, Hossain MS. Factors Associated with Misconceptions about HIV Transmission among Ever-Married Women in Bangladesh. Jpn J Infect Dis. 2015;68: 13–19. doi:10.7883/yoken.JJID.2013.323
