# Supplementary material for: Community’s misconception about COVID-19 and its associated factors in Satkhira, Bangladesh: A cross-sectional study
Source: PLoS One. 2021 Sep 10;16(9):e0257410. doi: 10.1371/journal.pone.0257410 (PMC8432838; doi:10.1371/journal.pone.0257410)
Supplement: Attachment — Submitted filename: Comments for Author.docx [file pone.0257410.s002.docx]

**Constructive Comments for Author(s)**

The topic is very interesting and timely issues that contribute for the tackling of the pandemic of COVID-19.

In order to make your manuscript to the standards of scientific community in sharing knowledge and experiences of your work with other scientific or researchers the following points are forwarded.

The topic “Community’s misconception about COVID-19 and its associated factors: Evidence from a cross-sectional study in Bangladesh”

**Better to make like this** “Community’s misconception about COVID-19 and associated factors in Satkhira, Bangladesh: a cross-sectional study”

Check grammars/spellings error carefully

On abstract methods part elaborate how you make selection of your sample

COVID-19: write in full words for the first time particularly in the Abstract

Mentions the level of confidences and measure of association (95% CI: AOR/OR)

Multistage sampling was employed why not use design effect?

On justification for conducting the study you state “lack of study in Bangladesh on the issue of misconception about COVID-19” add other reasons while this not enough.

**Introduction:** re writes and limits to 2 pages the introduction

**Operational definition for Community’s misconception**: give clear definition based on your classification categories after using ‘**PCA**’ not on each statement(s) response. Consult how to do PCA/computing different variables to generate those having and/not having misconception. What is your cut off points to say this **%** of individuals and over all scores should be computed to classify as _or + /have and/do not have misconception.

Which one you’re using median or mean score? Write clearly on this part

**Result**

Result: put the number then in bracket the %, like this N^o^ (%)

“Table 1. Household characteristics of the study population” give full name for the table (time and place)

Other focus areas of previous studies have been knowledge and awareness about COVID-19 among the general population [22], health professionals [23, 24], parents [25] and students [26] and vulnerable and susceptible populations [27–29]**.rewrite these paragraph** put the reference in single bracket it’s from page 3

Look at On page 10 “ Table 4. Unadjusted logistic analysis found **higher likelihood** of misconception among women (OR, 2.1, 95% CI, 1.16 - 3.77) compared with men.” Explain in this way Unadjusted logistic analysis found that the **likelihood** of misconception among women is 2times **higher than men counterpart**(OR, 2.1, 95% CI, 1.16 - 3.77).

Unadjusted logistic analysis found that the **likelihood** of misconception among women is 2 times higher when compared with men (OR, 2.1, 95% CI, 1.16 - 3.77).

Figures should be attached at the end of document

**Discussion**

Similar findings were reported across the world, including America [9, 39], Saudi Arabia [31] Nigeria [33,40], Nepal [7], Ghana [41], Uganda [42] and Pakistan [43].rewrite and put references in single bracket

Better if you avoid ‘we’ and use other terms like researchers ….in discussion mention study that also oppose /against your study finding, don’t only use the supportive one.

Conclusion:

Moreover, this study data was collected from only one district and three unions, therefore the findings may not be geralizable for the whole population of Bangladesh though the district and unions included were selected randomly.

Abstract: Conclusion we found that many people living in rural parts of Bangladesh have one or more misconceptions about COVID-19.we obtained a very higher percentage of the rural people of Bangladesh having one or more misconceptions related to COVID-19. Why you conclude for rural people of Bangladesh? Your study was from 210 respondents from three unions of Satkhira District out of 78 unions or 1distric out of 68 districs. So, reconsider your conclusions and make narrow the scope particular to your target populations/target geographic area.
