# Supplementary material for: Community’s misconception about COVID-19 and its associated factors in Satkhira, Bangladesh: A cross-sectional study
Source: PLoS One. 2021 Sep 10;16(9):e0257410. doi: 10.1371/journal.pone.0257410 (PMC8432838; doi:10.1371/journal.pone.0257410)
Supplement: Attachment — Submitted filename: Response to the reviewers.docx [file pone.0257410.s003.docx]

**Reviewers’ comments and author’s responses**

| **Reviewers’ comments** | **Author’s response** |
| --- | --- |
| **Reviewer #1** |  |
| 1. Write up and language need to revisit and proof read adequately by authors, if needed advised to take assistance from native speakers | We have now revised the manuscript thoroughly to improve the readability and grammar. It has also been reviewed by a native English speaker. |
| 2. Authors methodology is not fully clear and replicable | We have revised the methods section to make it fully replicable. |
| 3. Some of the result sections are not very clear and some selection seems biased on authors judgement | We have revised the results section to address the issue raised. Please have a look at the revised version. |
| **Specific** |  |
| 1. Section, Bakcground, P1, L5, check punctuation error “pandemic. [1].” | Thank you to bring this to our attention which we have now corrected. |
| 2. Section, Bakcground, P1, Please update the data based on current report “Bangladesh is one of the countries worst hit by the COVID-19 pandemic. More than a half million people has been infected among which around 9,000 people were died as per the official estimates of the World Health Organization for Bangladesh [15].” | As suggested, we have updated the relevant data. The updated sentence reads-  *Around 0.8 million people has been infected as of June 26, 2021 and around 14 thousands of which have died according to WHO’s estimates for Bangladesh* |
| 3. Method section “Primary data was collected during the period April 15 to May 09, 2020.” Which indicates study has been carried out long back during initial phase of pandemic and now already peak of second wave is ongoing so these findings may be not fully applicable to current scenario, which need to be discussed in limitation regarding cautious interpretation of the findings. | We agree with the reviewer that the issue addressed is important to be mentioned as the study limitation. We therefore added sentences now which read.  *Besides, the data was collected during the first wave of COVID-19 in Bangladesh, and therefore the findings are representative of the misconception that were common at the first wave of COVID-19. Bangladesh has been through multiple waves of the of the pandemic. However, these study findings are still valid and important for policy implications.* |
| 4. Method section, Data collection subsection: why not written consent?? “Verbal consent was taken from each respondent before collecting the data.” | This study is conducted in 9 villages where people are mostly illiterate. Moreover, they often feel discomfort to provide written signature. Therefore, we have taken verbal consent that also approved the broad provided ethical approval for this study considering the field level situation.  We have now added a few sentences to address this issue which read-  *Ethical review committee of the Institute of Biological Science, University of Rajshahi reviewed the study proposal and provided ethical approval. All participants were informed of the purpose of the study, the voluntary nature of the study, the anonymous use of the data and informed verbal consent was sought before data collection commenced.* |
| 5. Method section, Dependent variable, “These were COVID-19 is: (i) a punishment from god, (ii) revenge of nature, (iii) a virus war, (iv) not a dangerous virus, (v) not a disease and (vi) not a pandemic.” Were these six only misconceptions there?? If so what is the source? IF nor why these were only taken for dependent variables? | The misconception regarding the COVID-19 are many, some are global and many are country specific. However, the misconceptions that are commonly present in Bangladesh were first summarized for this study through comprehensive review in the Bangladeshi newspaper and mass media. The items summarized were used in the feasibility survey and field level misconception that were found consistent with this summarized list will finally be considered in this study. Any addition at this stage will also be included. Therefore, this covered most of the misconceptions’ items spread out in Bangladesh, however, we agree that these may not cover all. Therefore, we think it would be better to mention this issue as study limitation that we have done in this revised version. The added sentence read-  *Besides, the data was collected during the first wave of COVID-19 in Bangladesh, and therefore the findings are representative of the misconception that were common at the first wave of COVID-19. Bangladesh has been through multiple waves of the of the pandemic. However, these study findings are still valid and important for policy implications.* |
| 6. Methods section: I could see no mention of ethical consideration and their details in the methods and how verbal consent is enough for this study? | This issue has been addressed in our response to question number 4.. |
| 7. Result section: Table 1, arbitrarily while reporting normally distributed data, it is supposed to use Mean(±SD) than Mean (SE), please check and do needful | As suggested, we have now presented standard error. |
| 8. Result section: Table 3, how misconception present or absent decided? There are six questions about misconceptions in table 2 and it is divided into binary variable in table | We have used six items to generate the misconception variables. Each of the item was dichotomous, where a respondent was given “0” for correct response (no misconception) and 1 for wrong response (having misconception). The score generate for each individual item was then summed to calculate the total score of a respondent. Following this the median score was computed and this was used to divided the respondents as having misconception (if the individual score equal to or higher than median) and having no misconception (if the individual score lower than the median).  This approached are widely used in similar studies. |
| 3. I could not get how it is derived? Seems like punishment from god taken as misconception for table 3, but it’s labeling is opposite than in table 2. If so based on what rest attributes were neglected? | We are sorry, we failed to get your point here. In table 2, the misconception item “punishment from god” is correctly reported, however in table 3 there is no mention on misconception item “punishment from god” rather this table present the socio-demographic and media related variables across level of misconception. No misconception item was presented specifically in this table. |
| **Reviewer #2** |  |
| upon checking your manuscript" " i found that the manuscript is very important and timely issues that contribute for the tackling of the pandemic of COVID-19/ that helps the prevention of COVID-19 pandemic through identifying the community misconception to propose future plan.  However, the following points need to be answered/addressed. In order to make your manuscript to the standards of scientific community in sharing knowledge and experiences of your work with other scientific or researchers the following points are forwarded. | Many thanks for your critical comments that we have addressed in this revised version. |
| The topic “Community’s misconception about COVID-19 and its associated factors: Evidence from a cross-sectional study in Bangladesh” Better to make like this “Community’s misconception about COVID-19 and associated factors in Satkhira, Bangladesh: a cross-sectional study” | As suggested, we have revised the manuscript title. |
| Check grammars/spellings error carefully | We have revised the manuscript thoroughly to improve the readability and correct the grammar. |
| On abstract methods part elaborate how you make selection of your sample | As suggested, we have provided details of sample selection procedure. The added sentence reads-  *Multistage sampling method was used to collect data (n=210) from three unions of Satkhira District, Bangladesh.* |
| COVID-19: write in full words for the first time particularly in the Abstract  Mentions the level of confidences and measure of association (95% CI: AOR/OR)  Multistage sampling was employed why not use design effect? | As suggested, we have provided the full form of covid-19 in its first use.  We have provided confidence interval of the OR and AOR.  We have now adjusted the design effect. |
| On justification for conducting the study you state “lack of study in Bangladesh on the issue of misconception about COVID-19” add other reasons while this not enough. Introduction: re writes and limits to 2 pages the introduction | We have revised this section and provided a broad justification for the conduct of this research.  The introduction section has been revised and the content is reduced substantially. |
| Operational definition for Community’s misconception: give clear definition based on your classification categories after using ‘PCA’ not on each statement(s) response. Consult how to do PCA/computing different variables to generate those having and/not having misconception. What is your cut off points to say this % of individuals and over all scores should be computed to classify as _or + /have and/do not have misconception. Which one you’re using median or mean score? Write clearly on this part | The median score was used as cutoff point to classify respondents as having misconception and no misconception.  For checking, we run PCA and found a consistent result with the score that we used. We therefore keep median score-based classification. |
| **Results** |  |
| Result: put the number then in bracket the %, like this No (%) | As suggested, we have updated the results section and the number and percentage. |
| “Table 1. Household characteristics of the study population” give full name for the table (time and place) | We have renamed the table title as following-  Table 1. Household characteristics of the study population in three villages of Satkhira, 2020 |
| Other focus areas of previous studies have been knowledge and awareness about COVID-19 among the general population [22], health professionals [23, 24], parents [25] and students [26] and vulnerable and susceptible populations [27–29]. rewrite these paragraph put the reference in single bracket it’s from page 3 | As suggested, we have provided the reference together in a single bracket. |
| Look at On page 10 “ Table 4. Unadjusted logistic analysis found higher likelihood of misconception among women (OR, 2.1, 95% CI, 1.16 - 3.77) compared with men.” Explain in this way Unadjusted logistic analysis found that the likelihood of misconception among women is 2times higher than men counterpart (OR, 2.1, 95% CI, 1.16 - 3.77). | As suggested, we have updated the way of presenting the results. |
| Unadjusted logistic analysis found that the likelihood of misconception among women is 2 times higher when compared with men (OR, 2.1, 95% CI, 1.16 - 3.77). | As suggested, we have updated the results section. |
| Figures should be attached at the end of document | We have now added Figures at the end of the manuscript. |
| **Discussion** |  |
| Similar findings were reported across the world, including America [9, 39], Saudi Arabia [31] Nigeria [33,40], Nepal [7], Ghana [41], Uganda [42] and Pakistan [43].rewrite and put references in single bracket | This issue has been addressed in response to your previous comment, all references have put within a single paragraph. |
| Better if you avoid ‘we’ and use other terms like researchers ….in discussion mention study that also oppose /against your study finding, don’t only use the supportive one. | As suggested, we have updated the way of referring throughout the manuscript. |
| **Conclusion:** |  |
| Moreover, this study data was collected from only one district and three unions, therefore the findings may not be geralizable for the whole population of Bangladesh though the district and unions included were selected randomly. | As suggested, we have changed the conclusion section. The edited sentence reads-  *Moreover, information was collected only from one district and three unions and therefore the findings may not be generalizable to the whole population of Bangladesh through the district and unions included were selected randomly.* |
| Abstract: Conclusion we found that many people living in rural parts of Bangladesh have one or more misconceptions about COVID-19.we obtained a very higher percentage of the rural people of Bangladesh having one or more misconceptions related to COVID-19. Why you conclude for rural people of Bangladesh? Your study was from 210 respondents from three unions of Satkhira District out of 78 unions or 1distric out of 68 districts. So, reconsider your conclusions and make narrow the scope particular to your target populations/target geographic area. | We have revised this sentence to address this issue. The revised sentence reads-  *This study obtained a very higher percentage of misconception about the COVID-19 among the respondents analysed.* |
