# Supplementary material for: Community’s misconception about COVID-19 and its associated factors in Satkhira, Bangladesh: A cross-sectional study
Source: PLoS One. 2021 Sep 10;16(9):e0257410. doi: 10.1371/journal.pone.0257410 (PMC8432838; doi:10.1371/journal.pone.0257410)
Supplement: Attachment — Submitted filename: The 2nd time Comments for Authors.docx [file pone.0257410.s004.docx]

**28/07/2021**

**Constructive Comments on Revised manuscript for Author(s) response**

Dear /sir, nice to hear from you. So far most of the Comments given for you previously were addressed/ responded correctly as expected from you that are great. I have few points to be corrected in order to make your manuscript water proof before publication is commenced. So, I’ll appreciate for your concern with regard to the current comments which was given below:-

The primary topic “Community’s misconception about COVID-19 and its associated factors: Evidence from a cross-sectional study in Bangladesh” the manuscript title was revised as it was suggested.

Community’s misconception about COVID-19 and its associated factors in Satkhira, Bangladesh: a cross-sectional study

**Result:** in writing up the result part write the number then in the bracket write the percentage (%), like this **N^o^ (%) e.g. This is incorrect ‘Most of the respondents (113, 53.81**%)’ should be written as more than half of the respondents 113(53.81%)

With regard to the comments given about the topic was well addressed that is fine.

**New comment** Abstract part on methodology the last line particularly need to correct it: Univariate and multivariate logistic regression models were used to determine the factors associated with COVID-19 misconception 0.33 (95% CI: 0.13-0.84)...

Introduction and methodology parts were well addressed as per the comments given to them

Introduction parts were written to less than 2page, and multistage sampling was stated briefly

Response to each statement was reported dichotomously, “Yes (1)” *or* “No

(0)”. Aggregating these recoded responses, the misconception variable had been generated with

two categories, respondents who had scored greater than and equal to median were considered having high level of misconception and vice-versa. **The scoring was stated clearly well addressed**

**Conclusion: The summary was concluded /corrected as the provided** **comments but add the new comment.**

This study obtained a very higher percentage of misconception about the COVID-19 among the respondents **analysed**. This could be a potential challenge to fight against this pandemic which is now ongoing. Prioritizing mass and social media to disseminate evidence-based information as well as educate people about this disease is necessary.

**Results**

Around two-thirds (139, 66.19%) were males. **Most** of the respondents (113, 53.81%)

On Page 8, Table 3. Misconception were reported by higher proportion of men (74, 74.65%) than women (25, 25.25%). 70.27% (78) of the respondents who do not read Newspaper had a high level of misconception whereas 87.88% (**87**) of…correct as the e.g. given here for all upon writing the results. **New comment:-**

Table 1. Household characteristics of the study population in three villages of Satkhira, 2020

**Discussion**

e.g. Similar findings were reported across the world, Including America, Saudi Arabia, Nigeria, Nepal, Ghana, Uganda and Pakistan [8, 10, 32, 34, 40–44]. The comments given about discussion were correctly responded. **New comment**:-**Check reference N^o^ 33**
