# Supplementary material for: Community’s misconception about COVID-19 and its associated factors in Satkhira, Bangladesh: A cross-sectional study
Source: PLoS One. 2021 Sep 10;16(9):e0257410. doi: 10.1371/journal.pone.0257410 (PMC8432838; doi:10.1371/journal.pone.0257410)
Supplement: Attachment — Submitted filename: Response to the reviewers.docx [file pone.0257410.s005.docx]

**First reviewer:**

**Comment:** Many issues seems to be addressed, however labelling of table still has MD(SE), instead I assume that need to be MD(SD) as the value has changed!

Rest may be ok, however for potential errors as mentioned earlier, it is advised to proof read and finalize for needful ached.

**Our response:** We thank the reviewer to review our manuscript again and bring the typo in our attention which has corrected now. No other change is required.

**Constructive Comments on Revised manuscript for Author(s) response**

**Comment:** Dear /sir, nice to hear from you. So far most of the Comments given for you previously were addressed/ responded correctly as expected from you that are great. I have few points to be corrected in order to make your manuscript water proof before publication is commenced. So, I’ll appreciate for your concern with regard to the current comments which was given below:-

**Our response:** We thank the reviewer to review our manuscript for the second time. We would like to mention, we have carefully addressed your added comments during current revision.

**Comment:** The primary topic “Community’s misconception about COVID-19 and its associated factors: Evidence from a cross-sectional study in Bangladesh” the manuscript title was revised as it was suggested.

Community’s misconception about COVID-19 and its associated factors in Satkhira, Bangladesh: a cross-sectional study

**Our response:** Thanks. No further work is required here.

**Comments:**

**Result:** in writing up the result part write the number then in the bracket write the percentage (%), like this **N^o^ (%) e.g. This is incorrect ‘Most of the respondents (113, 53.81**%)’ should be written as more than half of the respondents 113(53.81%)

**Our response:** We have corrected the number formatting issue throughout the manuscript. The sentence mentioned has also revised as per the reviewer’s suggestion.

**Comment:** With regard to the comments given about the topic was well addressed that is fine.

**Our response:** Nothing need to be performed here.

**Comment:** Abstract part on methodology the last line particularly need to correct it: Univariate and multivariate logistic regression models were used to determine the factors associated with COVID-19 misconception 0.33 (95% CI: 0.13-0.84).

**Our response:** We have revised the sentence which now reads**-**

*Bivariate and multivariate logistic regression models were used to determine the factors associated with COVID-19 misconception.*

**Comment:** Introduction and methodology parts were well addressed as per the comments given to them

Introduction parts were written to less than 2 page, and multistage sampling was stated briefly

Response to each statement was reported dichotomously, “Yes (1)” *or* “No (0)”. Aggregating these recoded responses, the misconception variable had been generated with two categories, respondents who had scored greater than and equal to median were considered having high level of misconception and vice-versa. **The scoring was stated clearly well addressed**

**Our response:** No change is required here.

**Conclusion: The summary was concluded /corrected as the provided** **comments but add the new comment.**

This study obtained a very higher percentage of misconception about the COVID-19 among the respondents **analysed**. This could be a potential challenge to fight against this pandemic which is now ongoing. Prioritizing mass and social media to disseminate evidence-based information as well as educate people about this disease is necessary.

**Our response:** As suggested, we have revised this section which now read-

*This study obtained a very higher percentage of misconception about the COVID-19 among the respondents of Satkhira district in Bangladesh. This could be a potential challenge to fight against this pandemic which is now ongoing. Prioritizing mass and social media to disseminate evidence-based information as well as educate people about this disease is necessary.*

**Comments:**

**Results**

Around two-thirds (139, 66.19%) were males. **Most** of the respondents (113, 53.81%)

On Page 8, Table 3. Misconception were reported by higher proportion of men (74, 74.65%) than women (25, 25.25%). 70.27% (78) of the respondents who do not read Newspaper had a high level of misconception whereas 87.88% (**87**) of…correct as the e.g. given here for all upon writing the results. **New comment:-**

Table 1. Household characteristics of the study population in three villages of Satkhira, 2020

**Our response:** As suggested, we have corrected the number formatting issue throughout the manuscript and corrected the table title.

**Comment:**

**Discussion**

e.g. Similar findings were reported across the world, Including America, Saudi Arabia, Nigeria, Nepal, Ghana, Uganda and Pakistan [8, 10, 32, 34, 40–44]. The comments given about discussion were correctly responded. **New comment**:-**Check reference N^o^ 33**

**Our response:** The formatting issue of the reference 33 has corrected. No other change is required here.
